# Efficacy of probiotics on digestive disorders and acute respiratory infections: a controlled clinical trial in young Vietnamese children

**DOI:** 10.1038/s41430-020-00754-9

**Published:** 2020-09-28

**Authors:** Truong Tuyet Mai, Pham Thi Thu, Hoang Thi Hang, Tran Thi Thu Trang, Shintaro Yui, Akira Shigehisa, Vu Thuy Tien, Truong Viet Dung, Phan Bich Nga, Nguyen Trong Hung, Le Danh Tuyen

**Affiliations:** 1 National Institute of Nutrition, 48B Tang Bat Ho Street, Hanoi, Vietnam; 2 Haiphong University of Medicine and Pharmacy, 72 A, Nguyen Binh Khiem, Ngo Quyen, Haiphong Vietnam; 3 Yakult Central Institute, 5-11 Izumi, Kunitachi, Tokyo, 186-8650 Japan; 4 Yakult Honsha European Research Center for Microbiology ESV, Technologiepark 4, 9052 Zwijnaarde, Belgium; 5 Yakult Vietnam Co., Ltd., 195, Truong Van Bang Street, Thanh My Loi Ward District 2, Ho Chi Minh, Vietnam; 6 Hanoi Medical University, 1, Ton That Tung, Dong Da, Hanoi, Vietnam

**Keywords:** Malnutrition, Policy and public health in microbiology

## Abstract

**Objectives:**

To evaluate the efficacy of fermented milk containing *Lactobacillus casei* strain Shirota (LcS) on the incidence of constipation, diarrhea, acute respiratory infections (ARI), and nutritional status of young Vietnamese children.

**Methods:**

A controlled field trial was conducted with 1003 children (3–5 years old) in Thanh Hoa province in Vietnam. The probiotic group (*n* = 510) consumed fermented milk 65 mL/day containing 10^8^ CFU/mL of LcS for the 12-week intervention period, whereas the control group (*n* = 493) was not given any. The incidence of constipation, diarrhea, ARI, and anthropometry in children was determined at baseline, after 4, 8, and 12-week intervention, and after the 4-week follow-up period.

**Results:**

Probiotic drink decreased the incidence of constipation after the 12-week intervention period (12.0% vs. 32.0%, OR = 0.28 (95% CI: 0.21–0.40), *p* < 0.001), tended to decrease the incidence of diarrhea (4.9% vs. 7.9%, OR = 0.60 (95% CI: 0.35–1.01), *p* = 0.068), and prevented the occurrence of ARI (15.9% vs. 24.5%, OR = 0.58 (95% CI: 0.42–0.79), *p* < 0.001), when compared with the control group. In contrast, no probiotic effects were observed for the duration of diarrhea or ARI. Weight gain was higher in the probiotic group than in the control group after 4, 8, and 12-week intervention and after the 4-week follow-up period (*p* < 0.05).

**Conclusions:**

Daily intake of fermented milk containing LcS strongly prevented the incidence of constipation and ARI in Vietnamese children. This study also revealed the potential effects of the use of a probiotic drink on diarrhea prevention as well as nutritional status improvement.

## Introduction

The aim of the Global Millennium Development Goals and the new Sustainable Development Goals is to reduce health problems and mortality in children under 5 years of age [1]. The World Health Organization (WHO) reported that acute respiratory infection (ARI) is the leading cause of death in children, accounting for one-third of deaths that occur in the world each year [2]. This remains equally challenging in Vietnam, where the cause of death attributed to ARI ranges from 10 to 20% [3]. Diarrhea is the second leading cause of death in children, killing around 1.2 million susceptible children under 5 years of age due to dehydration and deteriorating nutrition status [2]. Constipation is also a common digestive disorder in children. The incidence of functional constipation in children ranges from 0.7 to 29% in both developed and developing countries with risk factors such as psychological stress, disturbed eating habits, and child maltreatment [4]. According to WHO, malnutrition in children under 5 years of age is concentrated in Asia and African countries. In Vietnam, despite many achievements in malnutrition prevention, the rate of malnutrition is elevated, and the stunted growth in children continues (24.6% in 2015) [5]. Malnutrition results in ARI, diarrhea, and physical and mental retardation in children [6].

The term probiotic, initially used in the 1960s, originates from a Greek word that means “for life”. It is currently defined as “live microorganisms that, when administered in adequate amounts, confer a health benefit to the host” [7]. Probiotics are widely used as components of dietary supplements or food products. However, the probiotic effect tends to be specific to a strain; therefore, the benefit of a strain should not be generalized [8–10]. *Lactobacillus casei* strain Shirota (*L. casei* YIT 9029; LcS) is a well-known probiotic strain that has been used commercially for a long time to produce fermented milk. Various aspects of the effects of LcS have been studied. Survival of LcS in the gastrointestinal tract has been validated in clinical trials conducted all over the world including Vietnam [11–13] along with its beneficial effects on constipation [14, 15], diarrhea [16, 17], and ARI [18, 19]. Moreover, while the impact on malnutrition has not been studied, one might expect beneficial effects of LcS on the nutritional status of children based upon reports from previous studies; for instance, preventing gastrointestinal dysfunction caused by infectious microorganisms or inducing short-chain fatty acids by commensal and probiotic bacteria in the intestines that can be used as an energy source by the host [20].

Herein, we conducted a controlled field trial to investigate the effects of a probiotic fermented milk product containing LcS on gastrointestinal symptoms, respiratory infections, and the nutrition status of children from Vietnam. This study is significant because, to the best of our knowledge, it is the first study in Vietnam with a large number of children investigating the multiple effects of LcS.

## Materials and methods

### Study design

The present study was approved by the Ethical Committee at the National Institute of Nutrition, Ministry of Health Vietnam, and conducted in accordance with the code of ethics of the World Medical Association (Declaration of Helsinki). The study was registered with ClinicalTrials.gov (NCT04346576).

This study was a community based, controlled open trial with or without fermented milk containing LcS in Vietnam children. The study was conducted over a period of 18 weeks consisting of 2-week screening/pre-observational period; 12-week intervention period; and 4-week post-intervention period.

### Sample size rationale

The sample size was estimated with a two-sided alpha of 0.05 and a power of 80%. In our preliminary survey in 6 communities in Thanh Hoa Province, the incidence rate of constipation was 28% (unpublished data). Previous studies reported an incidence rate of 20% in the probiotic group [14, 21], but that in the Control group remained unchanged following a 12-week intervention period. Based on this information, the minimum required sample size was estimated to be 443 participants per group. To allow for a drop-out rate of 10%, 492 children per group (total 984 children) were required from the pool of subjects participating in the screening phase and fulfilling the inclusion criteria described below.

### Inclusion/exclusion criteria

Nutrient-deprived children, which are at high risk for diarrhea and constipation, of either sex aged 3‒5 years were eligible for screening. The nutritional status was assessed using standard anthropometric measurements such as weight, height, and mid-upper arm circumference. A physician performed a thorough physical examination to determine the suitability of potential participants. All children were kept free from fermented milk product diets such as yogurt or cheese for 18 weeks including 2 weeks before intervention to rule out intake of lactic acid bacteria from any source other than the probiotic in our study. Children with neurological disorders, using long-term antibiotics, congenital chronic diseases such as congenital heart disease and congenital chronic kidney failure were excluded. Moreover, siblings were excluded to avoid affecting the results of ARI and diarrhea.

### Study population

One thousand six hundred and ninety-one nutrient-deprived children (3–5 year age) at six communities in Thanh Hoa, the Northern-most province in central Vietnam and the region with higher incidence of malnutrition (18.2%) and stunted growth (28.4%) according to the National Institute of Nutrition (2015) [5], were screened. The prevalence of malnutrition/ underweight, stunting, constipation, diarrhea, respiratory infection/cough, and runny nose, and economic status were investigated. Four communities were selected by considering the level of the economic and social conditions. The four communities were divided into Control group (two communities) and Probiotic group (two communities) according to the prevalence of underweight, stunting, constipation, diarrhea, respiratory infection/cough. Two boys, who are siblings, were excluded to reduce the risk of introducing bias in the results of ARI and diarrhea. Finally, 1036 children (518 children in the Control group and 518 children in the Probiotic group) were enrolled in this study. Informed consent signed by the guardians was obtained prior to the intervention initiation.

All children were kept free from other fermented milk products such as yogurt or cheese during the study.

### Subject management

A total of 1036 children (518 for each group) were monitored daily by central supervisors. In each community, 2‒3 central supervisors were assigned and supported 20‒25 local supervisors (teachers and parents/guardians) who were assigned for regular surveillance activities.

At baseline (T0), after 4 (T4), 8 (T8), 12 (T12) weeks of intervention and at the end of the 4-week follow-up period (T16), anthropometric data, logged dairy record, and health status were evaluated by a team of 15‒18 researchers including one pediatrician. For this evaluation, the team visited each community at three-day intervals. All survey/assessment activities were prepared and organized by local staff including staff from the Preventive Medicine Center, School teachers, Health staff, and other collaborators.

Teachers at kindergartens or guardians at home started to give the test products to the children in the Probiotic group throughout the 12-week intervention period, whereas children in the Control group were not given any. They completed a daily logbook for each child during the study period to check the intake of test products, defecation status, abdominal symptoms, other ROME III criteria items, ARI-related symptoms, dietary record, and other adverse events. For accurate monitoring, the researchers at the National Institute of Health carefully trained guardians and teachers to properly complete the logbook. Further, central supervisors checked the daily completed logbooks. Doctors responded appropriately to any unusual signs.

Functional constipation defined by ROME III [22] was evaluated every 4 weeks. It was diagnosed by doctors with reference to the logbook records. The stool consistency was recorded in a daily logbook based on the Bristol Stool Form Scale [23]. Stool consistency and number of defecations were evaluated as an average for the 4 weeks in each time point (Supplementary Tables S1 and S2).

Diarrhea and ARI were also evaluated by doctors every 4 weeks. Incidence of these diseases was assessed based on the logbook records from 2 weeks prior to each time point, whereas their duration was evaluated by the logbook records throughout the intervention period. Diarrhea was defined as more than three abnormally loose or liquid stools within a 24-h period. If there were at least 3 diarrhea-free days between a first and a second episode of diarrhea in the same individual, the second diarrhea episode was considered new. Children were diagnosed with ARI when they had 3 of the following 5 symptoms; fever, cough, runny nose, difficulty breathing, and increased breathing (≥40 times per minute). If all the symptoms of ARI were not observed for two consecutive days, the episode of ARI was considered to be completed.

Additionally, body weight and height were measured every 4 weeks by using electronic scales (TANITA, Japan) with accuracy up to 0.1 kg, and by the vertical height meter with accuracy up to 1 mm, respectively. The child’s nutritional status was assessed through anthropometric data collection according to the WHO 2006 Growth Standard. WHO AnthroPlus Software was used to evaluate the nutritional status of children to calculate the *Z*-score of an individual (Supplementary Table S3).

### Product handling

Probiotic test product was fermented milk containing 6.5 billion of LcS per 65 mL (10^8^ CFU/mL), manufactured by Yakult Vietnam, Co., Ltd. The nutritional composition of the test product is 0.8 g of protein, <0.1 g of fat, and 12.4 g of carbohydrates, and the total energy is 52.7 kcal per bottle. Subjects were asked to drink one bottle of the test product per day after lunch for 12 weeks on consecutive days. During the ingestion period, supervisors visited the subjects every day to give them one bottle of the test product.

Product pack was labeled in accordance with applicable laws and regulations. Each pack contained a label and an instruction sheet in the local language. The test products were stored in a refrigerator (<10°C), protected from direct sunlight, and used before their expiration date. Adherence to the intervention protocol was confirmed as follows: teachers at kindergartens or guardians at home provided the probiotic drinks (1 bottle/day × 7 days/week) after lunch. Supervisors also monitored intake of the test product.

### Study compliance

Test product consumption, intake of other fermented dairy products, and concomitant medications were recorded in the daily logbook to check the study compliance. The study team periodically reviewed the logbooks together with the subjects and reinforced the compliance.

### Statistical analysis

Statistical analyses were carried out using appropriate parametric and non-parametric tests and the statistical software program for social science (STATA 12, USA). Numerical data were presented as mean ± SD. Comparison of continuous variables was conducted with the Student’s *t*-test for normally distributed data. Appropriate tests were conducted for the data that were not normally distributed. Categorical data were compared using the Chi-square test. Paired *t*-test or McNemar test was applied for intra-group comparison. Statistical significance was set at a probability level of 0.05. Odds ratio (OR) and 95% confidence interval (95% CI) were calculated for each outcome as necessary.

## Results

Thirty-three children were excluded from analyses due to absence from one of the 4-week assessments. Thus, we used the data obtained from the remaining 510 children in the Probiotic group and 493 children in the Control group (totaling 1003 participants) for analyses. All participants in the Probiotic group consumed test products, over 95% of the time, during the 12-week intervention period.

### The characteristics of the study subjects

Baseline characteristics of the 1003 eligible participants are described in Table 1. There were no sex differences between the two groups. However, the ages of the children and their mothers in the Probiotic group were lower than those of the Control group (*p* < 0.05). Education level of mothers was not different between the two groups. Incidence of constipation, diarrhea, and ARI also did not show differences between the groups. However, the body weight and height of the children in the Probiotic group were lower than those of children in the Control group.

**Table 1:** Baseline characteristics of participants.

	Probiotic group (*n* = 510)	Control group (*n* = 493)
Sex (*n*, %)		
Male	274 (53.7)	284 (57.6)
Female	236 (46.3)	209 (42.4)
Age (month)	51.7 ± 10.0^*^	54.1 ± 8.6
Age of mothers (years old)	37.7 ± 11.7^*^	39.2 ± 12.8
Education level of mothers (*n*, %)		
<High school	261 (51.2)	272 (55.2)
≥High school	249 (48.8)	221 (44.8)
Constipation (*n*, %)	136 (26.7)	129 (26.2)
Diarrhea (*n*, %)	37 (7.3)	41 (8.3)
ARI (*n*, %)	118 (23.1)	123 (24.9)
Body weight(kg)	15.12 ± 2.82^*^	15.77 ± 2.93
Height (cm)	99.89 ± 6.93^*^	101.49 ± 6.10

*p < 0.05 compared to the Control group (Student *t*-test).

### Preventive effects of probiotics on constipation

**Fig. 1:**
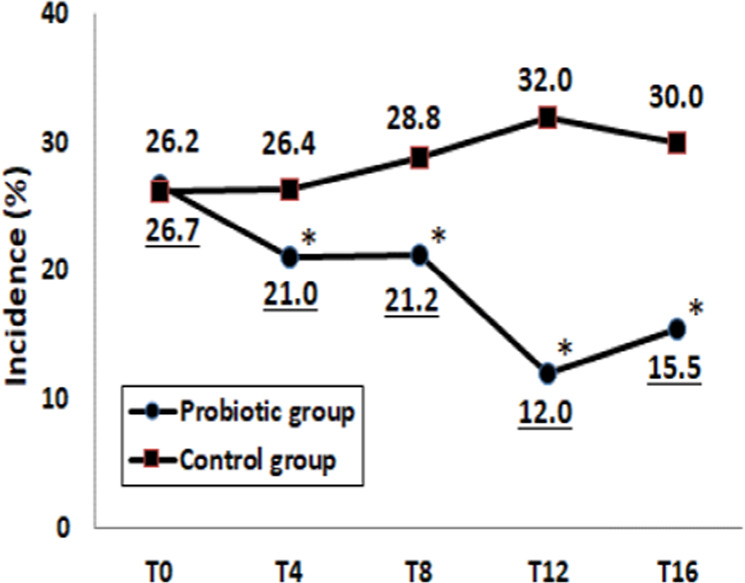
Incidence of constipation between Control and Probiotic groups. The incidence of constipation was monitored at baseline (T0), during intervention (T4–T12) and after 4-week follow-up (T16) in Control and Probiotic groups. *p < 0.05 compared to the Control group (Chi-square test).

The effects of probiotics on the incidence of constipation are shown in Fig. 1. The incidence of constipation was significantly lower in the Probiotic group than in the Control group at T4, T8, T12, and T16 (*p* < 0.05). Within the Probiotic group, the incidences of constipation at T12 and T16 decreased remarkably (*p* < 0.05 as vs. T0). The OR for the incidence of constipation after 12 weeks of intervention was 0.28 (95% CI: 0.21‒0.40, *p* < 0.001).

### Preventive effects of probiotics on diarrhea

**Fig. 2:**
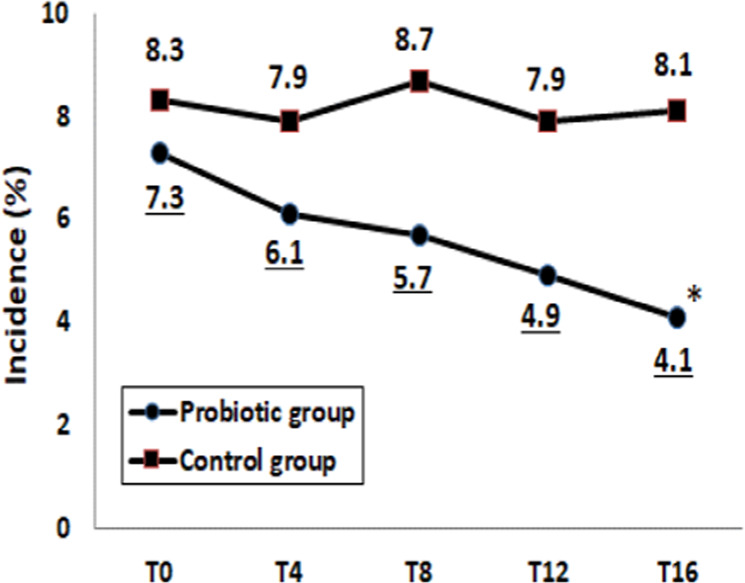
Incidence of diarrhea between Control and Probiotic groups. The incidence of diarrhea was monitored at baseline (T0), during intervention (T4–T12) and after 4-week follow-up (T16) in Control and Probiotic group. *p < 0.05 compared to the Control group (Chi-square test).

Effects of probiotics on the incidence of diarrhea are shown in Fig. 2. The incidence of diarrhea was significantly lower in the Probiotic group than in the Control group at T16 (*p* < 0.05). Within the Probiotic group, the incidence of diarrhea at T16 decreased remarkably (*p* < 0.05 as vs. T0). The OR for the incidence of diarrhea after 12 weeks of intervention was 0.60 (95% CI: 0.35‒1.01, *p* = 0.068).

### Preventive effects of probiotics on ARI

**Fig. 3:**
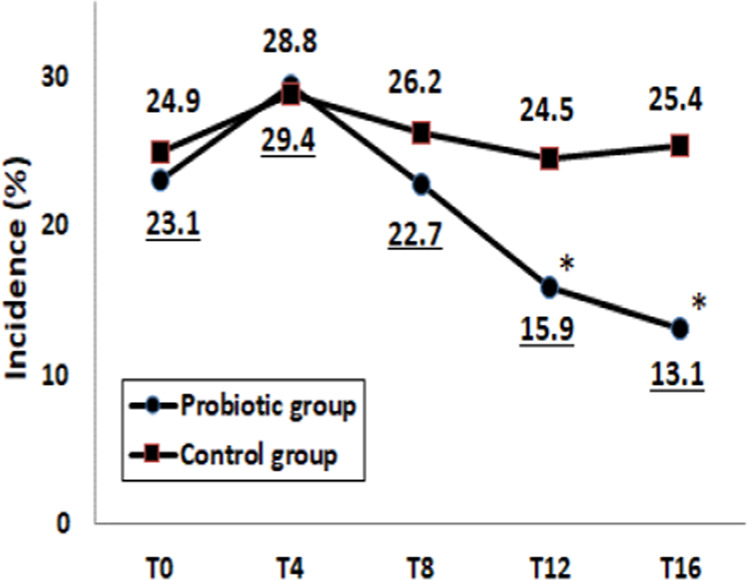
Incidence of ARI between Control and Probiotic groups. The incidence of ARI was monitored at baseline (T0), during intervention (T4–T12) and after 4-week follow-up (T16) in Control and Probiotic group. *p < 0.05 compared to the Control group (Chi-square test).

Effects of probiotics on the incidence of ARI are shown in Fig. 3. The incidence of ARI was significantly lower in the Probiotic group than in the Control group at T12 and T16 (*p* < 0.05). Within the Probiotic group, the incidence of ARI at T12 and T16 decreased remarkably (*p* < 0.05 as vs. T0). The OR for the incidence of ARI after 12 weeks of intervention was 0.58 (95% CI: 0.42‒0.79, *p* < 0.001).

### Effects of probiotics on the duration of diarrhea or ARI

Effects of probiotics on the duration of diarrhea or ARI are shown in Table 2. The intervention had no significant effect on the duration of both diseases.

**Table 2:** Average duration of diarrhea or ARI per episode reported from the start of the intervention (T0) to the end of the intervention (T12).

	Probiotic group	Control group
Average days of diarrhea/episode	4.8 ± 7.0	3.9 ± 5.0
Average days of ARI/episode	4.4 ± 4.3	5.0 ± 4.7

### Effects of probiotics on nutrition improvement

Effects of probiotics on body weight and height are shown in Table 3. From baseline (T0) to T16 of the study period, the means of body weight in the Probiotic group were lower than those in the Control group, except for T8 (*p* < 0.05). However, the changes in levels of body weight from baseline in the Probiotic group at each time point were greater than those in the Control group (*p* < 0.05). The mean height in the Probiotic group was also lower than that in the Control group (*p* < 0.05) from baseline (T0) to T16. The changes in height from baseline in the Probiotic group at T4, T12, and T16 were greater than those in the Control group (*p* < 0.05). At T8, the change in height of the Control group was greater than that of the Probiotic group (*p* < 0.05).

**Table 3:** Changes in the body weight (kg) and height (cm) during the 12-week intervention (T0–T12) and 4 weeks of follow-up (T16).

Time	Body weight (kg)		Height (cm)	
	Probiotic group	Control group	Probiotic group	Control group
T_0_	15.12 ± 2.82*	15.77 ± 2.93	99.89 ± 6.93*	101.49 ± 6.10
T_4_	15.36 ± 2.87*	15.86 ± 2.94	100.42 ± 6.92*	101.96 ± 6.09
T_8_	15.63 ± 2.89	15.98 ± 2.94	100.89 ± 6.93*	102.61 ± 6.09
T_12_	15.72 ± 2.90*	16.17 ± 2.98	101.57 ± 6.94*	103.06 ± 6.07
T_16_	15.87 ± 2.90*	16.33 ± 2.98	101.98 ± 6.92*	103.39 ± 6.02
Changes				
*T*_*4*_ –*T*_*0*_	0.23 ± 0.28*	0.09 ± 0.29	0.53 ± 0.27*	0.48 ± 0.27
*T*_*8*_ –*T*_*0*_	0.51 ± 0.38*	0.21 ± 0.39	1.00 ± 0.40*	1.13 ± 0.40
*T*_*12*_ –*T*_*0*_	0.60 ± 0.43*	0.39 ± 0.46	1.68 ± 0.46*	1.58 ± 0.48
*T*_*16*_*-T*_*0*_	0.75 ± 0.47*	0.55 ± 0.50	2.09 ± 0.51*	1.91 ± 0.59

*p < 0.05 compared to the Control group (Student *t*-test).

## Discussion

In this study, the administration of fermented milk containing 6.5 billion LcS per day for 12 consecutive weeks resulted in a significant reduction in the incidence of constipation in Vietnamese children. Many studies have documented the effect of LcS and showed that probiotics could attenuate constipation in human trials [14, 15, 24, 25]. Tilley et al. demonstrated that LcS improved the hardness of stools in adults with mild constipation [14]. The effect of LcS on constipation has been proven in elderly patients [24], women [15], and healthy adults [14, 25], although another study showed no reduction in the incidence or frequency of constipation [21]. This study is the first to demonstrate the effect of LcS on constipation in Vietnamese children aged 3‒5 years at the community level, which could enhance the universality of the benefit of LcS on constipation.

LcS was reported to improve diarrhea by improving and regulating the intestinal microbiota and environment [16, 17]. In this study, while LcS did not affect the duration of diarrhea, the incidence of diarrhea tended to be improved after 12 weeks of intervention, and the OR was 0.60 (*p* = 0.068). There are many studies on the use of probiotics (*Lactobacillus* or *Bifidobacteria*) for diarrhea or gastrointestinal disease treatment [26, 27]. Sur et al. conducted a double-blind, randomized, controlled trial with 3758 children aged 1‒5 years in an urban slum in Kolkata, India [16] and reported that daily LcS intake played a role in preventing acute diarrhea in young children in a developing country’s community environment. Wong et al. showed that LcS reduced the incidence of antibiotic-associated diarrhea in emergency room patients [28].

This study provides evidence that supports the benefits of LcS to prevent infectious diseases. ARI is also a major target of probiotics, and several studies around the world have demonstrated the usefulness of probiotic products for the improvement of ARI. Weizman et al. showed that infants who received *Bifidobacterium lactis* or *Lactobacillus reuteri* exhibited significantly fewer fever episodes [29]. Another study involving children aged 1‒3 years, who received formula milk with oligosaccharide and *B. lactis* HN019 for 1 year, found a reduction in the incidence of pneumonia, severe acute lower respiratory infections, and days of severe illness and high fever [30]. However, several studies have reported different results for the changes in immune status caused by different strains of probiotics, including both positive and negative results. Thus, it is important to accumulate data on the effect of probiotic strains on immune function or infectious diseases to verify their credibility. Recently, it was demonstrated that LcS reduced the frequency and duration of ARI in office workers [18] and exerted protective effects against infections in athletes [19, 31]. The mechanism underlying the immunoregulatory function of LcS was upregulation of NK cell activity [32]. In this study, we did not check NK cell activity, and thus, further investigation is required to identify this mechanism in Vietnamese children.

Some studies have shown that probiotics may improve the nutritional status of children [30, 33]. However, many factors affect the nutritional status, and the effectiveness of probiotics might be affected by the intervention period, sample size, and characteristics of study subjects. In our study, weight gain in the Probiotic group was significantly higher than that in the Control group after 12 weeks of intervention. Conversely, baseline age between the two groups was different, and significantly higher energy intake was observed in the Probiotic group after 12 weeks of intervention (data not shown, survey not for all subjects). Therefore, it is not clear whether the weight gain observed in the Probiotic group was due to a gap in age, the increase in energy intake, or the improvement of nutritional absorption by probiotics. Some studies have shown the protective effects of probiotics on malnourished children in terms of improved nutritional status, but further investigation is required.

### Limitations

Three limitations of this clinical study should be considered. First, there was a difference in background information between the groups, especially the age of the children. Though the incidence of constipation, diarrhea, and ARI did not differ between the groups at baseline, body weight and height were markedly different due to the age differences between the groups. Second, the double-blind placebo-controlled design was not adopted in this clinical trial, because this study also served as a basic health survey for the children in Thanh Hoa, Vietnam. Third, there is no accumulation of supportive data for the potential beneficial effect of LcS on children’s nutritional absorption. This needs to be verified in the future via in vitro and clinical studies.

## Conclusion

Habitual consumption of fermented milk containing LcS prevented constipation and ARI in Vietnamese children, and it may be useful for treating diarrhea and improving nutritional status, thus conferring remarkable health benefits to children in Vietnam.

## Supplementary information

Table S1, Table S2, Table S3

Table S1, Table S2, Table S3 legends
