# Supplementary material for: Efficacy of probiotics on digestive disorders and acute respiratory infections: a controlled clinical trial in young Vietnamese children
Source: Eur J Clin Nutr. 2020 Sep 28;75(3):513–20. doi: 10.1038/s41430-020-00754-9 (PMC7943424; doi:10.1038/s41430-020-00754-9)
Supplement: Supplementary file 1 — Table S1, Table S2, Table S3 [file 41430_2020_754_MOESM1_ESM.doc]

**Supplementary Information**

***Table S1. Changes in the stool consistency during the study in children
with constipation,*** at baseline (T0)

| Time | Probiotic group  (n = 136) | Control group  (n = 129) |
| --- | --- | --- |
| T0-T4 | 3.58 ± 0.51 * | 3.42 ± 0.62 |
| T4-T8 | 3.59 ± 0.57 | 3.49 ± 0.64 |
| T8-T12 | 3.67 ± 0.50 * | 3.41 ± 0.64 |
| T12-T16 | 3.75 ± 0.42 * | 3.42 ± 0.60 |

*Data were calculated as average values during the period based on the Bristol Stool Form Scale data in the logbook records.*

**p<0.05 compared to the Control group. (Student t-test)*

***Table S2. Changes in the defecation frequency per week during the study
in children with constipation, at baseline (T0)***

| Time | Probiotic group  (n = 136) | Control group  (n = 129) |
| --- | --- | --- |
| T0-T4 | 6.28 ± 2.05 * | 5.76 ± 2.05 |
| T4-T8 | 5.95 ± 1.82 | 5.48 ± 2.18 |
| T8-T12 | 6.01 ± 1.62 * | 5.44 ± 2.43 |
| T12-T16 | 6.18 ± 1.45 * | 5.31 ± 2.22 |

*Data were calculated as average values during the period based on the logbook record.*

**p<0.05 compared to the Control group. (Student t-test)*

***Table S3. Changes in the Z-Score (W/A) and Z-Score (H/A) during the 12-week intervention (T0-T12) and 4weeks of follow-***up (T16)

| Time | Z-Score (W/A) | |  | Z-Score (H/A) | |
| --- | --- | --- | --- | --- | --- |
|  | Probiotic group  (n = 510) | Control group  (n = 493) |  | Probiotic group  (n = 510) | Control group  (n = 493) |
| T_0_ | -0.94 ± 1.08 | -0.82 ± 1.10 |  | -1.20 ± 0.92 | -1.14 ± 0.88 |
| T_4_ | -0.90 ± 1.07 | -0.85 ± 1.10 |  | -1.21 ± 0.92 | -1.16 ± 0.88 |
| T_8_ | -0.84 ± 1.05 | -0.87± 1.10 |  | -1.22 ± 0.91 | -1.12 ± 0.87 |
| T_12_ | -0.86 ± 1.05 | -0.84 ± 1.10 |  | -1.17 ± 0.91 | -1.12 ± 0.87 |
| T_16_ | -0.86 ± 1.04 | -0.84 ± 1.09 |  | -1.18 ± 0.90 | -1.15 ± 0.86 |
| Changes |  |  |  |  |  |
| *T_4_ -T_0_* | 0.04 ± 0.15 | -0.04 ± 0.15 |  | -0.01 ± 0.06 | -0.00 ± 0.06 |
| *T_8_ -T_0_* | 0.10 ± 0.19 * | -0.05 ± 0.19 |  | -0.01 ± 0.09 | 0.02 ± 0.09 |
| *T_12_ –T_0_* | 0.08 ± 0.22 * | -0.03 ± 0.22 |  | 0.04 ± 0.11 | 0.03 ± 0.11 |
| *T_16_-T_0_* | 0.08 ± 0.23 * | -0.02 ± 0.24 |  | 0.02 ± 0.13 * | -0.00 ± 0.13 |

**p<0.05 compared to the Control group. (Student t-test)*
