# Supplementary material for: Efficacy of probiotics on digestive disorders and acute respiratory infections: a controlled clinical trial in young Vietnamese children
Source: Eur J Clin Nutr. 2020 Sep 28;75(3):513–20. doi: 10.1038/s41430-020-00754-9 (PMC7943424; doi:10.1038/s41430-020-00754-9)
Supplement: Supplementary file 2 — Table S1, Table S2, Table S3 legends [file 41430_2020_754_MOESM2_ESM.doc]

**Summary of Supplementary Information (Tables):**

Table S1. Changes in the stool consistency during the study in children who were constipated at baseline (T0). The stool consistency was assessed based on the Bristol Stool Form Scale data in the log book.

Table S2. Changes in the defecation frequency per week during the study period in children who were constipated at baseline (T0). Data were calculated as average values during the period based on the logbook record.

Table S3. Change in the Z-Score (W/A) and Z-Score (H/A) during the 12-week intervention (T0-T12) and 4-week follow-up (T16)

*Supplementary Information accompanies this paper on European Journal of Clinical Nutrition website. (http://www.nature.com/ejcn)*
